# Protective and risk physical activities for adolescent idiopathic scoliosis: a systematic review identifying one-hour daily activity threshold and Chinese school-based prevention framework

**DOI:** 10.3389/fspor.2025.1644314

**Published:** 2025-09-02

**Authors:** Yujie Guan, Bin Zhao, Yongchun Fan, Yunchao Li, Haozhe Wang

**Affiliations:** ^1^ The Second Clinical College, Heilongjiang University of Chinese Medicine, Harbin, Heilongjiang, China; ^2^ Department of Musculoskeletal Pain, The Second Affiliated Hospital of Heilongjiang University of Chinese Medicine, Harbin, Heilongjiang, China; ^3^ Department of Rehabilitation Medicine, The Second Hospital of Heilongjiang Province, Harbin, Heilongjiang, China; ^4^ College of Physical Education, China University of Mining and Technology, Xuzhou, Jiangsu, China

**Keywords:** adolescent idiopathic scoliosis, physical activity, health promotion, adolescent health, prevention

## Abstract

**Objective:**

To systematically review the association between different types and intensities of physical activity and adolescent idiopathic scoliosis (AIS) risk, synthesize evidence on dose-response relationships between physical activity and AIS prevention, and propose school intervention recommendations.

**Methods:**

Literature related to physical activity for preventing scoliosis and promoting spinal health in adolescents was retrieved from PubMed, Cochrane Library, Web of Science, Scopus, China National Knowledge Infrastructure, and Google Scholar, published between January 2015 and January 2025. After screening, key information was extracted, and methodological quality was assessed using the Newcastle-Ottawa Scale for case-control and cohort studies, the AHRQ methodology checklist for cross-sectional studies, and the Cochrane Risk-of-Bias tool and Jadad scale for randomized controlled trials. The protocol was registered with PROSPERO (CRD420251065070).

**Results:**

Twenty-one studies were included after screening, of good methodological quality, involving 77,813 adolescents, including 5,259 AIS patients, published between 2015 and 2025. Physical activity was significantly associated with AIS. Most recreational sports and traditional Chinese sports may prevent AIS, while long-term participation in competitive sports, dance, and artistic gymnastics were risk factors for AIS. Adolescents need to achieve a minimum threshold of one hour of daily physical activity to effectively prevent spinal scoliosis.

**Conclusion:**

Appropriate physical activity may reduce AIS risk. Schools should enhance their focus on adolescent physical activity and establish an integrated “school-family”, “school-medical”, and “school-sports” approach to support scoliosis prevention and promote healthy adolescent development.

**Systematic Review Registration:**

https://www.crd.york.ac.uk/, PROSPERO (CRD420251065070).

## 1 Introduction

Idiopathic scoliosis (IS) refers to an unexplained lateral curvature of the spine, a three-dimensional deformity (including curvature in the coronal plane, sagittal instability, and rotation in the horizontal plane), which accounts for approximately 80% of all types of scoliosis (1, 2). Adolescent idiopathic scoliosis (AIS) is the predominant form of IS, primarily observed in adolescents aged 10–18. Globally, the prevalence of AIS ranges from 0.5% to 5.2%, depending on the country and region (3). The total detection rate of spinal abnormalities among Chinese primary and secondary school students was 2.8%, and the prevalence rate of scoliosis was 1.2%. It has increased annually since 2008 and has become the third major threat affecting the physical and mental health of primary and secondary school students, following myopia and obesity (4). Though the etiology of AIS remains unclear, studies suggest associations with behavioral patterns, environmental factors, and school management. Some studies have shown that AIS patients have altered body composition, characterized by tall stature, low body weight, reduced bone density, and decreased muscle mass at puberty, which correlates with disease severity (5, 6).

AIS not only affects an adolescent's appearance, mobility, and mental health, but in severe cases can lead to cardiopulmonary dysfunction (7). Countries worldwide are actively exploring correlations between AIS and factors such as body mass index, behavioral patterns, posture, and lifestyle habits to develop effective prevention and control strategies. Some countries have incorporated “spinal screening” into routine medical check-ups for students, establishing a complete closed-loop management system of screening-referral-intervention (8–12).

Physical activity is defined as any physical movement in which energy is produced and expended by skeletal muscles (13). Physical activity improves body composition, maintains body fat balance, improves bone density, and promotes mental health (14). However, as modern lifestyles change, screen-based entertainment is gradually replacing physical activity, resulting in decreased physical activity time and negative impacts on children and adolescents (15). There have been studies that have attempted to explore the correlation between physical activity and AIS, but no consensus has been reached yet. The 2024 Chinese Guidelines for Rehabilitation and Treatment of Adolescent Idiopathic Scoliosis (16) recommend assessing physical activity capacity in AIS patients and planning appropriate sports activities for prevention.

Previous research suggests a potential association between physical activity and AIS, but the relationship between specific types of physical activity and AIS remains unclear. Therefore, we systematically analyzed the correlation between physical activity and AIS to lay the foundation for developing evidence-based physical activity interventions.

## 2 Materials and methods

This systematic review was conducted following the recommendations of PRISMA (17). Our review was registered in PROSPERO with the protocol CRD420251065070.

### 2.1 Search strategy

We systematically searched PubMed, Cochrane Library, Web of Science, Scopus, Google Scholar, and China National Knowledge Infrastructure (CNKI) from June 1, 2024, to January 15, 2025. We searched for articles on physical activity to prevent AIS and promote spinal health in adolescents, encompassing all available online publications. We conducted subject searches and title/abstract screening, restricting results to literature published between January 2015 and January 2025. The English keywords used in the search were “scoliosis”, “adolescent idiopathic scoliosis”, “physical exercise”, “physical activity”, among others. Table 1 shows the search terms and Table 2 shows the search strategies and results for different databases and search engines.

**Table 1:** Search terms.

“Scoliosis”	Operator	“Physical activity”
Scoliosis or adolescent idiopathic scoliosis or AIS or spinal deformity or spinal curvature	AND	Exercise or physical exercise or physical activity or aerobic exercise or isometric exercise or exercise training or Competitive sports

**Table 2:** Search strategy and results.

Databases	Search strategy	Results	Search time
PubMed (2015–2025)	((“Scoliosis”[Mesh]) OR ((((adolescent idiopathic scoliosis[Title/Abstract]) OR (AIS[Title/Abstract])) OR (spinal deformity[Title/Abstract])) OR (spinal curvature[Title/Abstract]))) AND (((((((exercise[Title/Abstract]) OR (physical exercise[Title/Abstract])) OR (physical activity[Title/Abstract])) OR (aerobic exercise[Title/Abstract])) OR (isometric exercise[Title/Abstract])) OR (exercise training[Title/Abstract])) OR (Competitive sports[Title/Abstract]))	480	2024.7.7–2025.1.15
Cochrane Library (2015–2025)	In Trials; Title Abstract Keyword: Scoliosis or adolescent idiopathic scoliosis or AIS or spinal deformity or spinal curvature AND exercise or physical exercise or physical activity or aerobic exercise or isometric exercise or exercise training or Competitive sports	958	2024.7.7–2025.1.15
Web of Science (2015–2025)	((TI = (Scoliosis or adolescent idiopathic scoliosis or AIS or spinal deformity or spinal curvature)) AND TI = (exercise or physical exercise or physical activity or aerobic exercise or isometric exercise or exercise training or Competitive sports)) AND PY = (2015–2025)	337	2024.7.7–2025.1.15
Scopus (2015–2025)	TITLE-ABS-KEY: (Scoliosis or adolescent idiopathic scoliosis or AIS or spinal deformity or spinal curvature) AND (exercise or physical exercise or physical activity or aerobic exercise or isometric exercise or exercise training or Competitive sports)	317	2024.7.7–2025.1.15
Google Scholar (2015–2025)	(Scoliosis or adolescent idiopathic scoliosis or AIS or spinal deformity or spinal curvature) AND (exercise or physical exercise or physical activity or aerobic exercise or isometric exercise or exercise training or Competitive sports)	50	2024.7.7–2025.1.15
CNKI (2015–2025)	SU: (Scoliosis OR Spinal curvature OR Spinal deformity OR Spinal abnormality OR Adolescent idiopathic scoliosis) AND (Physical activity OR Exercise OR Sports activities OR Influencing factors OR Movement OR Training OR Ba Duan Jin OR Yi Jin Jing OR Tai Chi)-In Chinese	379	2024.7.7–2025.1.15

### 2.2 Inclusion and exclusion criteria

Inclusion criteria were developed using the Population, Interventions, Comparators, Outcome, Study Designs (PICOS) qualification criteria described in PRISMA: (1) P: Adolescents aged 6–18 years diagnosed with AIS by imaging (Cobb angle ≥10 ˚) or clinical assessment (ATR ≥ 5˚); (2) I: Physical activity (recreational sports, competitive sports, traditional Chinese sports, school sports programs); (3) C: Different activity types, activity intensities, sedentary behavior, usual care; (4) O: AIS incidence, prevalence, progression; Cobb angle, angle of trunk rotation (ATR); physical activity levels and dose-response relationships; (5) S: Randomized controlled trials (RCTs), cohort studies, case-control studies, cross-sectional studies.

The exclusion criteria were as follows: (1) Full-text articles unavailable; (2) Repeatedly published studies; (3) Studies with incomplete data and of low quality; (4) Reviews, conference papers, or case reports.

### 2.3 Study selection and data extraction

Two researchers (Y.F. and Y.L.) independently selected literature using EndNote X9 software. Articles were first excluded based on title and abstract screening, followed by full-text review to determine final eligibility. Any disagreements during the selection process were resolved through consultation with a third researcher (B.Z.) to ensure literature alignment with the study objectives. Data extracted included: author, country, year, study type, population, objectives, physical activity type and intensity, scoliosis characteristics, and outcomes.

### 2.4 Methodological quality assessment

Two researchers (Y.G. and H.W.) independently assessed study quality, with disagreements resolved by a third researcher (B.Z.). The Newcastle-Ottawa Scale (NOS) (18) was used to assess case-control and cohort study quality. NOS assesses three domains across eight items (maximum 9 points): selection (4 points), comparability (2 points), and outcome assessment (3 points). The NOS scores are classified as low quality (0–3), moderate quality (4–6), or high quality (7–9). The Agency for Healthcare Research and Quality (AHRQ) methodology checklist (19) was used to score the quality of the cross-sectional studies. Studies were classified as low (0–3), moderate (4–7), or high quality (8–11). The Cochrane Risk-of-Bias Evaluation Tool for Randomized Controlled Trials was used to score the quality of RCTs. The Cochrane Risk-of-Bias Evaluation Tool (20) and Jadad scale (21) were used to assess the quality of RCTs. The Cochrane tool assesses risk of bias across seven domains: randomization process, deviations from intended interventions, missing outcome data, measurement of the outcome, selection of the reported result, and overall bias, with each domain rated as low risk, some concerns, or high risk of bias. The Jadad scale evaluates RCTs across five items (maximum 5 points): randomization (2 points), blinding (2 points), and withdrawals/dropouts (1 point). Jadad scores are classified as low quality (0–2) or high quality (3–5).

## 3 Results

### 3.1 Study selection

**Figure 1:**
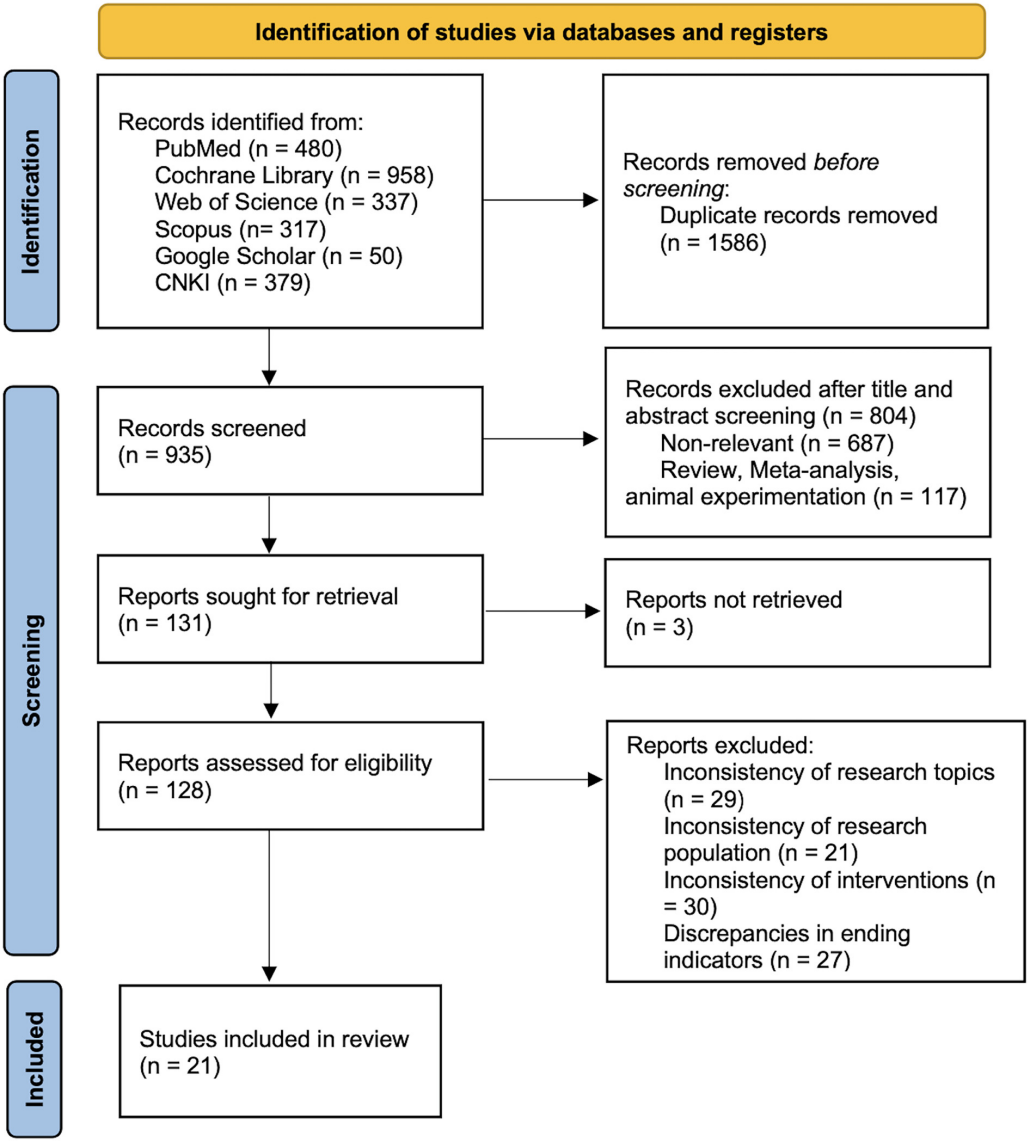
Systematic review and meta-analysis reporting standards (PRISMA 2020) literature screening.

Due to the excessive amount of irrelevant literature retrieved from the Web of Science database, it was decided to use a literature title search in this database in order to improve the accuracy of the search results. Initial searches identified 2,521 records from five databases and one search engine. After removing 1,586 duplicates, 935 titles and abstracts were screened, excluding 804 irrelevant studies. Following exclusion of 3 unretrievable reports, 128 full texts were assessed, with 21 studies meeting inclusion criteria. Figure 1 shows a flowchart of the selection process for the studies.

### 3.2 Study characteristics

The articles were from different countries and regions, including the USA (*n* = 1), France (*n* = 1), Japan (*n* = 1), Brazil (*n* = 1), Sweden (*n* = 1), Israel (*n* = 1), Croatia (*n* = 1), the UK (*n* = 2), Canada (*n* = 2), Italy (*n* = 3), and China (*n* = 7). The types of studies included cohort studies (*n* = 2), case-control studies (*n* = 5), cross-sectional studies (*n* = 11), and RCTs (*n* = 3). A total of 77,813 adolescents were involved, of whom 5,259 were diagnosed with AIS. Studies were published between 2015 and 2025. Studies primarily originated from preventive medicine, school health, rehabilitation medicine, and sports medicine. The basic characteristics of the included studies are shown in Table 3.

**Table 3:** Basic characteristics of included studies.

Included literature and year	Country	Study design	Sample characteristics	Diagnostic criteria	Activity type and intensity	Results
Recreational sports						
McMaster et al. (2015) (22)	Scotland	Longitudinal case-control	EG: AIS, *n* = 79, Cobb angle = 10–78 (female = 66, male = 13, mean age = 15.1) CG: *n* = 77(female = 66, male = 11, mean age = 14.7)	(1)	Swimming, dance, gymnastics, karate, ice skating, soccer, tennis, field hockey, horseback riding	AIS positively correlated with toddler indoor warm water swimming and independent toe-touching; negatively correlated with participation in recreational sports
Watanabe et al. (2017) (23)	Japan	Cross-sectional	2,600, 10–14years AIS, *n* = 1,228, Cobb angle ≥15˚	(2)	Ballet, basketball, badminton Starting age, years of participation, duration, frequency (1/week, 2–3/week, 4/week)	Ballet is a risk factor for AIS; basketball and badminton are protective factors
Tobias et al. (2019) (24)	UK	Prospective cohort	4,640, mean age = 15 AIS, *n* = 267, Cobb angle ≥10˚	(4)	Running, basketball, swimming etc. at age 10 Average frequency (<1, 1–3, ≥4 times/week) MVPA, LPA or sedentary time at age 11	Sedentary children have higher prevalence of scoliosis; physical activity may be a protective factor for AIS
Chopra et al. (2020) (25)	USA	Case-control	EG: AIS, *n* = 24(*n* = 12, Cobb <40˚; *n* = 12, Cobb ≥40˚) CG: *n* = 12	(2)	Four consecutive days of activity and sedentary behavior	Reduced activity and increased sedentary time are risk factors for AIS
Scaturro et al. (2021) (10)	Italy	Cross-sectional	428, 11–14 years, mean age = 11.7 AIS, *n* = 47, Cobb angle ≥10˚	(1)	Recreational sports, high-risk activities (dance or artistic gymnastics) Weekly activity time (≤3 h, >3 h)	High-risk sports with weekly duration ≥3 hours associated with AIS
Steinberg et al. (2021) (26)	Israel	Cross-sectional	132, 12–14 years AIS, *n* = 38, ATR ≥ 5˚	(1)	Dance	Fixed participation in dance classes shows high prevalence of AIS in adolescents
Negrini et al. (2023) (27)	Italy	Retrospective cohort	AIS, *n* = 511 (female = 415, male = 96, mean age = 11.9 ± 1.2)	(2)	Recreational sports	Recreational sports have a protective effect on adolescent spinal health and can delay AIS progression
Zain et al. (2016) (32)	Italy	Cross-sectional	Tennis group: *n* = 102 (male = 50, female = 52) Same-age group: *n* = 203 (male = 101, female = 102) AIS, *n* = 33, ATR ≥ 5˚, ATR ≥ 7˚	(1)	Tennis	Tennis as a unilateral sport does not increase AIS risk
de Assis et al. (2021) (28)	Brazil	Retrospective case-control	EG: AIS, *n* = 78 CG: *n* = 78	(1)	Physical activity active, irregularly active, sedentary	Insufficient physical activity and the classification of schoolchildren as irregularly active were regarded as risk factors for scoliosis
Competitive Sports						
Zaina et al. (2015) (33)	Italy	Cross-sectional	112, mean age = 12.5 AIS, ATR ≥ 5˚	(1)	Competitive swimming	Competitive swimming increases the risk of AIS
González-Ruiz et al. (2025) (29)	Canada	Cross-sectional	343, 7–18 years AIS, *n* = 11, ATR ≥ 5˚	(1)	Competitive swimming, volleyball, rugby	Competitive swimming, volleyball and rugby are risk factors for AIS
Traditional Chinese Sports						
Luan et al. (2024) (37)	China	RCT	EG: *n* = 30 CG: *n* = 30	(1)	YiJinJing Twelve Postures 12 weeks	YiJinJing Twelve Postures can effectively prevent AIS
Yao et al. (2021) (38)	China	RCT	EG: *n* = 8 CG: *n* = 8 AIS, ATR: 4˚-7˚	(1)	Tai Chi 12 weeks	Tai Chi can improve balance ability in AIS patients
Fang et al. (2018) (39)	China	RCT	EG: *n* = 15 CG: *n* = 15	(2)	YiJinJing 30 min daily, 3 months	YiJinJing can improve spinal mobility and prevent AIS
School Sports						
Zhao et al. (2023) (9)	China	Cross-sectional	18,399, 12 years AIS, *n* = 368, Cobb angle ≥10˚	(2)	Weekly PE classes: 1, 2, 3, 4, ≥5	Average 3 or 4 PE classes per week negatively correlated with AIS in 12-year-old students; lower AIS prevalence in those participating in sports interest classes
Zhang et al. (2023) (34)	China	Cross-sectional	27,926, 7–15 years AIS, *n* = 1,067, Cobb angle ≥10˚	(2)	Daily sedentary time <10 h, ≥10 h Daily physical activity ≥1 h, <1 h	<1 h daily physical activity, ≥10 h daily sedentary time, and sports with unilateral force are risk factors for AIS
Yu et al. (2023) (35)	China	Cross-sectional	1,674, primary, middle and high school AIS, *n* = 113, Cobb angle ≥10˚	(2)	Daily outdoor activity >2 h, ≤2 h	Daily outdoor activity time ≤2 h is a risk factor for AIS
Huang et al. (2024) (36)	China	Cross-sectional	1,732, primary, middle, high and vocational high school AIS, *n* = 84, Cobb angle ≥10˚	(2)	Daily outdoor activity <1 h, 1-<2 h, 2-<3 h, ≥3 h	Daily outdoor activity time 1 to <2 h associated with lower AIS risk
Glavaš et al. (2023) (11)	Croatia	Cross-sectional	18,216, 11, 12, 14 years AIS, *n* = 1,053	(1)	Weekly school physical activity, PE classes <3 h, ≥3 h	Physical activity time <3 h/week is a risk factor for AIS; AIS patients have lower physical activity levels
No Impact						
Gaume et al. (2020) (30)	France	Case-control	EG: AIS, *n* = 19 (*n* = 9, untreated; *n* = 10, brace treatment) CG: *n* = 25	(3)	Daily walking distance, weekly physical activity intensity	AIS does not affect adolescents’ exercise levels
Diarbakerli et al. (2016) (31)	Sweden	Case-control	EG: AIS, *n* = 239 G: *n* = 58	(2)	Vigorous activity, high-intensity exercise	Physical activity has little impact on AIS prevalence

AIS, adolescent idiopathic scoliosis; EG, experimental group; CG, control group; RCT, randomized controlled trial; MVPA, moderate to vigorous physical activity; VPA, vigorous physical activity; Cobb angle, indicator of scoliosis severity; Adams forward bend test, Widely used scoliosis screening method; ATR, angle of trunk rotation, (1): Adams forward bend test and ATR, (2): Full spine x-ray, (3): EOS imaging, (4): Full spine DXA scan imaging.

### 3.3 Literature quality assessment

Among the included studies, studies (11, 22–31) were of high quality, studies (9, 10, 32–36) were of moderate quality, and the three RCTs (37–39) had a low risk of bias and high quality. Detailed quality assessment results are provided in Supplementary Materials (SupplementaryTables S1–S3 and Supplementary Figures S1–S2).

### 3.4 Assessment of physical activity

Two of the 21 studies (24, 25) used accelerometers to measure physical activity intensity. One prospective cohort study (24) collected parent-recalled activity data at 18 months and child self-reported activity data at 10 years to analyze the relationship between physical activity content and AIS. Another study (25), a cross-sectional case-control study, used the SRS-22 questionnaire to clinically assess patients. A cross-sectional case-control study (31) used the International Physical Activity Questionnaire Short Form (IPAQ-SF) to assess the level of physical activity, as well as the proportion of moderate-intensity physical activity in daily life. A retrospective case-control study (28) used both the IPAQ-SF and the Competitive Sports Practice Questionnaire (CSPQ) to examine the correlation between physical activity levels and AIS. A cross-sectional case-control study (30) used a smartphone pedometer and self-reporting to collect daily walking distance and physical activity intensity from study participants. A longitudinal case-control study (22) interviewed mothers and adolescents about regular physical activity during preschool years and after-school/weekend activities. A retrospective cohort study (27) examined the rate of progression of scoliosis by assessing whether and how often participants exercised. Three RCTs (37–39) assessed the association between traditional Chinese sports and AIS through comparative clinical comparisons. The remaining studies were cross-sectional (9–11, 23, 26, 29, 32, 33, 34–36), which assessed the type, frequency, and duration of physical activity through observational methods and questionnaires.

### 3.5 Physical activity content and outcomes

Of the 21 studies, 19 demonstrated that physical activity is associated with AIS and showed a certain dose-response relationship. To make the subsequent analysis clearer, this paper categorizes the included literature according to common ways of categorizing physical activities: recreational sports (10, 22–28), competitive sports (29, 33), traditional Chinese sports (37–39), and school sports (9, 11, 34–36).

#### 3.5.1 Recreational sports

McMaster et al. (22) collected data on the physical activities of the study participants before and after the age of 5 years through interviews. The study found higher AIS prevalence among those who swam in heated indoor pools as toddlers and could touch their toes independently. In contrast, those who participated in dance, gymnastics, karate, ice skating, soccer, and field hockey after the age of 5 had a lower prevalence of AIS. Watanabe et al. (23) recorded participants’ time and frequency in different sports and found that adolescents in classical ballet training were more prone to AIS. AIS prevalence increased with training frequency, experience years, and training duration. However, playing basketball and badminton was inversely associated with the prevalence of AIS, suggesting that these two physical activities may prevent the development of AIS. Tobias et al. (24) collected the activity levels of the study participants at 18 months and 10 years of age through parent and child recall. The researchers also measured the exercise levels of the study participants at 11 years using accelerometers. The results showed that infants who stood independently at 18 months were 66% less likely to develop scoliosis in adolescence. Children physically active at 10 years (basketball, swimming, running, etc.) were 53% less likely to develop scoliosis. Those regularly engaging in moderate-to-vigorous physical activity at 11 years were 30% less likely to develop scoliosis. Chopra et al. (25) used four triaxial accelerometers to measure the daily physical activity time and sedentary time of the study participants over four consecutive days. The study found that the higher the severity of scoliosis, the shorter the physical activity time and the longer the sedentary time of the patients. This suggests that AIS may reduce patients’ exercise capacity and produce adverse health effects in the long term. Scaturro et al. (10) conducted a cross-sectional study in 11 secondary schools in Italy. AIS was associated with ≥3 hours of high-risk exercise (dance, artistic gymnastics) per week. Steinberg et al. (26) focused on the effects of prolonged dance exercise on the spine. Young girls who regularly participated in dance classes showed a high prevalence of AIS. Negrini et al. (27) examined the long-term benefits of physical activity on adolescent spinal health. The study found that long-term regular physical activity can slow AIS progression. Zaina et al. (32) studied the relationship between tennis and AIS. Tennis as a unilateral sport did not increase AIS risk. de Assis et al. (28) explored the relationship between physical activity levels and AIS. The study concluded that sedentary behavior and low physical activity are risk factors for AIS.

#### 3.5.2 Competitive sports

Zaina et al. (33) conducted a study with competitive swimmers. These athletes underwent prolonged, intense swimming training, resulting in asymmetrical stress on spinal muscles. The study suggested that competitive swimming increases AIS risk. González-Ruiz et al. (29) concluded that competitive swimming, volleyball, and rugby are all risk factors for the development of AIS. These sports involve movement patterns and training intensities that place high stress on the spine, disrupting normal spinal growth and increasing AIS prevalence.

#### 3.5.3 Traditional Chinese sports

Traditional Chinese sports Luan et al. (37) used twelve YiJinJing postures as an exercise intervention to investigate their efficacy in preventing AIS and improving spinal posture. The results showed that YiJinJing could protect spinal health and prevent AIS. Yao et al. (38) explored the effects of Tai Chi practice on subjects’ spinal balance ability and demonstrated that Tai Chi was beneficial in enhancing spinal stability. Fang et al. (39) trained subjects in YiJinJing for 30 minutes daily over three months, which significantly improved spinal mobility and helped prevent spinal curvature.

#### 3.5.4 School sports

Glavaš et al. (11) conducted a study with secondary school students. The study found that physical activity <3 hours/week was associated with AIS onset. Additionally, AIS patients demonstrated slightly lower physical activity levels than healthy controls. The remaining four studies (9, 34–36) were conducted in Inner Mongolia Autonomous Region, Henan Province, Guangdong Province, and Hubei Province, China. These studies explored the relationship between physical activity and AIS during campus scoliosis screening. Zhao et al. (9) found that attending an average of 3–4 physical education classes per week and having participated in physical and aesthetic specialty classes were negatively associated with the development of AIS. Zhang et al. (34) found that <1 hour of daily physical activity, ≥10 hours of daily sedentary time, and predominantly unilateral power sports were risk factors for scoliosis in primary and secondary school students. Yu et al. (35) found that ≤2 hours of outdoor activity per day was a risk factor for the development of AIS. Huang et al. (36) found that primary and secondary school students with physical activity <1 hour per day had a lower risk of scoliosis than those with 1 to <2 hours of physical activity per day. These twelve studies demonstrated correlations between physical activity and AIS from multiple perspectives through rigorous analysis. The findings suggest that a comprehensive understanding of the relationship between physical activity and AIS is essential for effective AIS prevention.

#### 3.5.5 Differences in study results

Two studies reported a weak association between physical activity and AIS.

Diarbakerli et al. (31) concluded that physical activity was not significant in preventing AIS. However, the study focused on comparing participants’ higher-level physical activities, which included both strenuous sports and amateur high-level activities. Since study subjects engaged in high-level activity while typical adolescents engage in low-to-moderate activity, this may explain the similar activity levels between patients and controls. This suggests a dose-response relationship in physical activity for AIS prevention, where medium to high levels of physical activity showed no significant preventive effect. This suggests that future research should focus more on the relationship between low to moderate levels of physical activity and AIS.

Gaume et al. (30) focused on whether AIS affects people's level of physical activity. They concluded that AIS patients have similar physical activity levels to healthy individuals. However, using smartphone pedometers for physical activity measurement introduced bias due to non-standardized cell phone models, placement methods, and pedometer applications, as well as the small sample size (*n* = 44).

## 4 Discussion

Before discussing our findings, we clarify the terminology used throughout this review. Physical activity is defined as any bodily movement produced by skeletal muscles that requires energy expenditure, encompassing daily activities, exercise, and sports. Sports refer to organized, competitive physical activities with established rules, ranging from recreational to competitive levels. Exercise represents structured, repetitive physical activity aimed at improving fitness. Our analysis categorizes evidence according to activity context and intensity rather than strict definitional boundaries, as the included studies used varying terminologies.

### 4.1 Relationship between AIS and physical activity programs

Based on a systematic analysis of 21 studies, we classified the relationship between different types of physical activity and the risk of AIS. Table 4 summarizes the key findings related to protective factors and risk factors in different categories of physical activity.

**Table 4:** Summary of physical activity types and their relationship with AIS.

Physical activity category	Specific activities	Relationship with AIS	Supporting studies	Key findings
Recreational Sports				
	Basketball	Protective	(23, 24)	Lower AIS prevalence
	Badminton	Protective	(23, 27)	Inversely associated with AIS prevalence
	Soccer	Protective	(22, 24)	Lower AIS risk in participants
	Tennis (recreational)	Protective	(32)	No increased AIS risk as unilateral sport
	Ice sports	Protective	(22, 27)	Lower AIS prevalence
	Karate	Protective	(22, 27)	Lower AIS prevalence
	Field hockey	Protective	(22)	Lower AIS prevalence
	Swimming (recreational)	Protective	(23, 24)	Non-competitive swimming prevents AIS
Risk activities	Classical ballet/dance	Risk factor	(10, 23, 26)	Dose-response relationship; ≥3 h/week increases risk
	Artistic gymnastics	Risk factor	(10)	High-risk activity when ≥3 h/week
Competitive sports				
	Competitive swimming	Risk factor	(29, 33)	Asymmetrical spinal stress from intense training
	Competitive volleyball	Risk factor	(29, 47)	Repetitive asymmetric loads
	Competitive rugby	Risk factor	(29)	High spinal biomechanical overload
Traditional Chinese Sports				
	YiJinJing	Protective	(37, 39)	Improves spinal posture and mobility
	Tai Chi	Protective	(38)	Enhances balance and spinal stability
	Baduanjin	Protective	(48)	Improves spinal mobility
Unilateral Sports				
	NA	Mixed results	(11, 23, 32, 34)	Conflicting evidence; needs further research

#### 4.1.1 Recreational sports

Several studies consistently show that dance or artistic gymnastics increases AIS risk (40–42). Studies by Watanabe et al. (23), Scaturro et al. (10), and Steinberg et al. (26) support this finding. Adolescents who participated in classical ballet and artistic gymnastics training were at higher risk of developing AIS. This association shows a clear dose-response relationship: as the frequency, years, and intensity of training increase, the incidence and severity of AIS increase. This may be due to several reasons: (1) Dance and artistic gymnastics populations are predominantly female, with low body weight and high stature. These characteristics are all AIS risk factors (5, 43, 44). (2) To pursue aesthetics and movement coordination, dancers and artistic gymnasts often perform movements that disrupt spinal physiological curvature, including generalized joint hypermobility (GJH), which leads to decreased spinal stability. Overstretched muscles and soft tissues cannot effectively support the spine, resulting in scoliosis (45). (3) Dancers and artistic gymnasts tend to train from childhood. Repeated spinal hypermobility during growth and development can place a greater load on the spine and interfere with normal spinal development (26). Although the study by McMaster et al. (22) included dance as a protective factor for AIS, this study had limitations including a small dancer population and short-term follow-up, leading to low confidence in the conclusions. Therefore, although there is uncertainty about the specific association of dance or artistic gymnastics with AIS, excessive engagement in dance- and artistic gymnastics-type physical activities increases the risk of AIS. This population requires focused monitoring and targeted protection.

For other types of recreational sports, studies (22–24) suggest that basketball, badminton, soccer, tennis, ice sports, karate, and field hockey can be used as preventive physical activities for AIS. These physical activities prevent AIS through multiple mechanisms, including: regulating adolescent psychology, improving anxiety and depression (46), improving neuromotor control, exercising the muscles around the spine, and stabilizing the trunk and pelvis. For swimming, McMaster et al. (22) found that early exposure to heated swimming pools in infants increases adolescent AIS risk. Studies by Watanabe et al. (23) and Tobias et al. (24) on non-competitive swimming showed that swimming can prevent AIS by increasing muscle strength around the spine and maintaining spinal stability. However, current cross-sectional studies on recreational sports have limited scope and credibility, necessitating large-scale prospective studies to determine specific physical activity-AIS relationships.

#### 4.1.2 Competitive sports

Competitive sport aims to enhance athletic performance through high-intensity repetitive specialized training. Three papers have suggested that competitive swimming (29, 33), volleyball (47), and rugby (29) increase AIS risk due to muscular-skeletal imbalance from fixed training patterns that trigger spinal biomechanical overload. Repetitive asymmetric loads from sport-specific movements cause paraspinal muscle fatigue, uneven vertebral growth plate stress, and structural scoliosis adaptation. Importantly, current studies are cross-sectional, which limits causal inference. Future prospective cohort studies or RCTs are needed to validate the correlation between competitive sport training patterns and AIS prevalence.

#### 4.1.3 Traditional Chinese sports

Traditional Chinese sports, including Baduanjin (48), YiJinJing (37, 39), and Tai Chi (38), offer potential AIS prevention benefits through unique mechanisms that improve spinal stability via symmetrical movement patterns, progressive core training, and neuromuscular control. The effect of the exercise intervention is mainly reflected in three aspects: (1) Correcting postural spinal imbalance through multidimensional symmetrical movements in the sagittal, coronal, and horizontal planes; (2) Strengthening the deep core muscles by combining dynamic and static training to build a spinal stability support system; (3) Improving proprioception and motor control through synergistic regulation of respiration, movement, and intention, based on the traditional Chinese medicine theory of “unity of form and spirit”. Based on current research, we suggest establishing a three-tier intervention system tailored to adolescent growth and development characteristics. This system includes exercise dosage (frequency/intensity/duration), standardized movement assessment, and individualized progressive programs. Additionally, large multi-center longitudinal studies are needed to verify long-term intervention effects.

#### 4.1.4 Unilateral sports

Conventional wisdom suggests that unilateral movement may increase the risk of AIS through movement imbalance and abnormal postural control. The findings of two studies (11, 34) support this view. A significant increase in the prevalence of AIS was found among those who regularly engaged in unilateral sports. However, studies by Watanabe et al. (23) and Zaina et al. (32) concluded that playing tennis four or more times a week was not associated with the development of AIS. Badminton can also be used as a protective sport for AIS. Recent longitudinal evidence has provided important new insights into this debate. Bonavolontà et al. (49) conducted a six-month longitudinal study of 44 university athletes, comparing the effects of symmetrical exercises (running, cycling) with asymmetrical exercises (tennis) on the spine. The results showed that only two of 25 spinal parameters exhibited statistically significant differences between groups, with small effect sizes that were not clinically relevant. This study suggests that in non-professional athletes, the impact of moderate asymmetric exercise loads (within 4–5 hours per week) on the spine may be overestimated, suggesting that moderate asymmetric sport practice can be safely encouraged without exceeding recommended weekly training volumes. The literature shows conflicting views on whether unilateral sports increase the risk of AIS, and this issue remains unresolved. Current evidence suggests that exercise volume may be more important than the symmetry of exercise type, but more high-quality prospective studies are needed to clarify the exact relationship between unilateral sports and AIS.

### 4.2 Relationship between AIS and physical activity intensity

#### 4.2.1 Physical activity intensity thresholds

Four studies (24, 25, 28, 34) reported that sedentary behavior is a risk factor for AIS by affecting adolescents’ cardiorespiratory fitness, exercise capacity, and potentially causing psychological problems. Two studies (10, 23) demonstrated that dance and artistic gymnastics are AIS risk factors, with severity positively correlated with high-risk sport intensity. Although the optimal physical activity intensity threshold for AIS prevention remains undetermined, Zhang et al. (34) and Huang et al. (36) concluded that <1 hour of daily physical activity increases AIS risk. This is consistent with Yu et al. (35), who found that AIS prevalence was 1.87 times higher in adolescents with <1 hour vs. ≥1 hour of daily physical activity. The World Health Organization 2020 Guidelines (13) and Chinese Physical Activity Guidelines (50) both recommend ≥60 minutes of daily physical activity for adolescents. In summary, to effectively prevent AIS, the minimum threshold of daily physical activity intensity needs to be 1 hour, and the optimal threshold still needs to be determined by future research. However, longer duration of physical activity is not always better, and overactivity may produce pain, muscle tension in the low back, excessive disc pressure, and spinal instability (51).

#### 4.2.2 Changes in physical activity capacity

AIS patients may experience a decrease in physical activity capacity. Although smartphone pedometer measurements (30) showed unaffected daily walking distance in AIS patients, more objective accelerometer measurements (25) revealed reduced daily walking distance and physical activity capacity compared to non-AIS adolescents, consistent with other studies (11, 52). This may be related to body asymmetry in patients with AIS, which leads to abnormalities in balance, coordination, breathing patterns, and gait, decreased control during physical activity, and a higher probability of pain and sports injuries. Therefore, AIS patients may have reduced physical activity abilities and require additional guidance to participate appropriately in physical activities for improved fitness and spinal health protection.

### 4.3 The interrelationship between AIS and physical activity

There is an important interrelationship between AIS and physical activity, but research in this area remains insufficient and requires further exploration. Most studies primarily focused on how physical activity influences AIS onset and progression, revealing that different types and intensities have varying effects. Protective physical activity (such as recreational sports (22–24, 27, 32), Baduanjin (48), YiJinJing (37, 39), and Tai Chi (38) has shown preventive effects, while high-risk physical activity (such as competitive sports (29, 33, 47), dance (10, 23, 26), and artistic gymnastics (10) increases AIS risk. Research indicates that daily physical activity lasting at least one hour is the minimum threshold for preventing AIS. However, evidence on how AIS affects adolescent participation in physical activity remains limited. Only two studies (11, 25) have addressed this relationship, showing that AIS severity negatively correlates with daily physical activity time and positively correlates with sedentary behavior, and that AIS patients have slightly lower activity levels than healthy individuals. These studies suggest that AIS may influence adolescents’ motor abilities and participation through multiple mechanisms. From a biomechanical perspective, three-dimensional spinal deformity alters trunk mechanical properties. This affects core stability and dynamic balance, limiting athletic performance and endurance. Additionally, spinal deformity may cause pain during sustained physical activity, leading to avoidance behaviors that reduce participation. Psychosocial factors also play a significant role, as AIS patients often experience body image concerns and reduced self-confidence, further diminishing their willingness to participate in group activities. These negative interactions may form a vicious cycle: AIS reduces physical activity levels → insufficient daily activity fails to meet the one-hour threshold → muscle strength and cardiorespiratory function decline → AIS progression → Further decline in physical activity levels, ultimately impacting adolescents’ overall health and quality of life. Therefore, understanding this bidirectional relationship mechanism has important clinical implications for developing personalized AIS management strategies and exercise intervention programs.

This interrelationship is also reflected in proprioceptive performance. Notarnicola et al. (53) revealed an important phenomenon: AIS patients generally have abnormal perceptions of their own scoliosis. Among sedentary patients, 34.8% were completely unaware of their scoliosis, while among patients who participated in physical activities, this proportion dropped to 17.5%. This suggests that physical activity can increase proprioceptive input and body spatial cognition in AIS patients, enhancing their ability to perceive spinal curvature. However, it does not improve their accurate perception of the deformity's specific characteristics. Additionally, AIS patients often feel dissatisfied with their appearance and exhibit attentional bias toward deformed areas (54). Research (53) also shows that physical activity participation may increase patients’ concern about their spinal deformity, potentially creating psychological burden. Therefore, while physical activity enhances body perception, it may also reinforce appearance anxiety related to spinal deformity in AIS patients, creating a complex dual effect. This finding has important implications for clinical practice: when recommending physical activity for AIS patients, it is essential to consider not only its biomechanical benefits but also its potential psychological impacts. Clinicians and exercise instructors should help patients develop accurate body cognition, avoid excessive focus on physical defects, and strengthen psychological support and health education to ensure that physical activity promotes mental and physical well-being. Future research should explore how to minimize the negative impact of physical activity on the mental health of AIS patients while maintaining its physiological benefits.

### 4.4 Prevention of AIS by physical activity in the school perspective

Schools serve large populations of adolescents. School-based AIS prevention programs can create healthy environments that promote spinal health awareness and good habits among students. Globally, adolescents in all countries generally suffer from high learning pressure, long sitting time, and short physical activity time, which puts the spine in potential danger (55–58). Schools must therefore develop multidimensional physical activity prevention systems to reduce AIS incidence, beyond their traditional educational role (11, 59).

Internationally, several authorities have proposed AIS prevention strategies. The 2016 guidelines of the International Society of Scoliosis Orthopaedic Treatment (SOSORT) emphasized the importance of physical activity in the prevention of AIS (8). The United States Preventive Services Task Force (USPSTF) recommends school screening programs as a means of early detection of AIS (3). The United Kingdom implements a multidisciplinary prevention and control program that focuses on cross-sectoral collaboration (24). Italy and Spain have established a complete “screening-referral-intervention” system for AIS (8). In Australia, the spinal health promotion program integrates a prevention system that combines school physical education curriculum reform, family education, and supervision by medical institutions (60).

Based on current evidence, we suggest that schools should ensure students have at least 1 hour of daily physical activity (34, 36) and 3–4 physical education periods per week (9, 11). We propose an innovative multidimensional prevention system combining “school-family”, “school-medicine”, and “school-sport” integration, which incorporates international prevention strategies while addressing China's specific context to form a comprehensive prevention approach.

**Figure 2:**
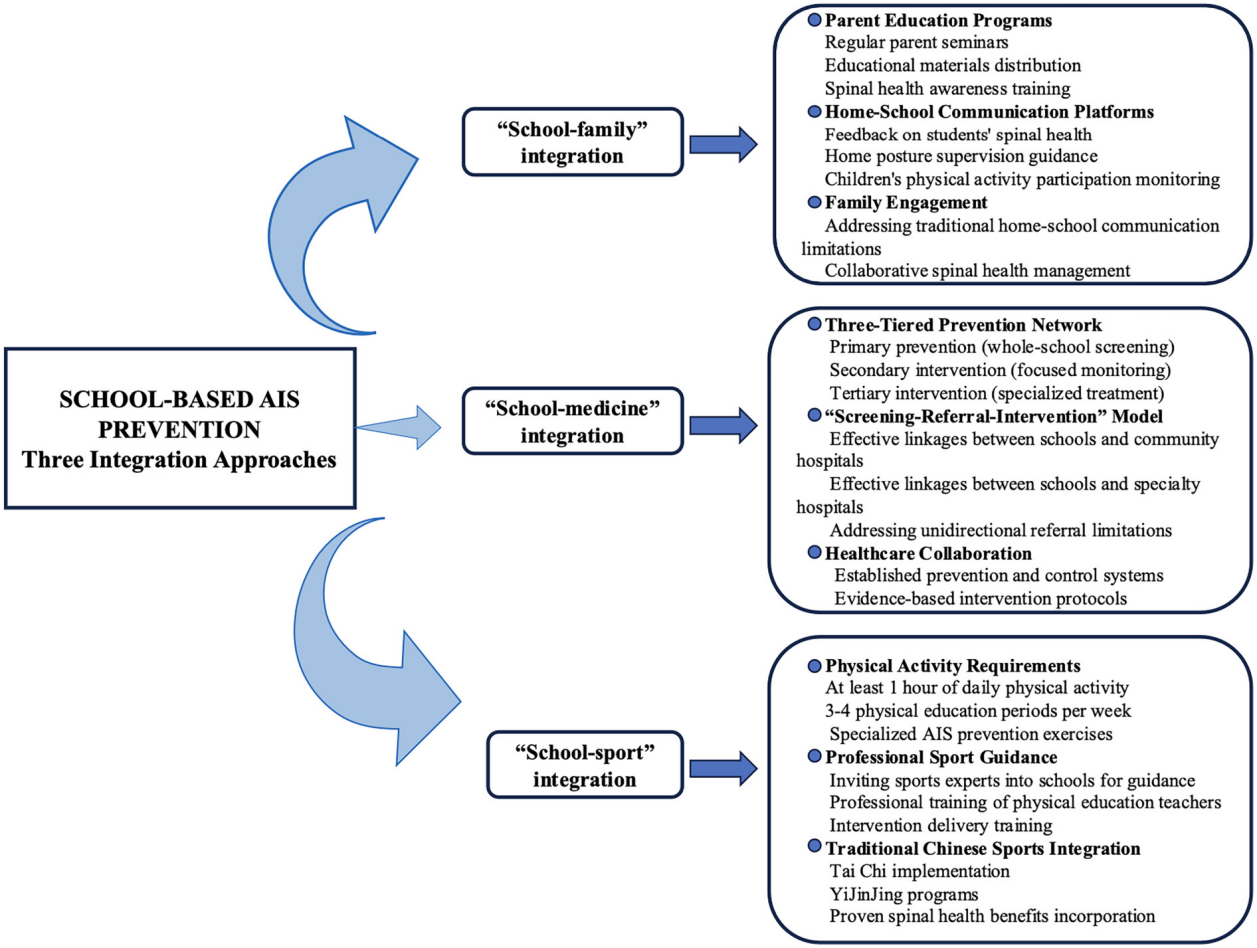
Prevention of AIS by physical activity in the school vision.

(1) “School-family” integration: Schools organize regular parent seminars, distribute educational materials, establish home-school communication platforms, provide feedback on students’ spinal health, and guide parents in supervising children's posture and physical activity participation at home, addressing traditional home-school communication limitations (60). (2) “School-medicine” integration: Based on the European “screening-referral-intervention” model (8), we propose a three-tiered school-medicine prevention network: primary prevention (whole-school screening), secondary intervention (focused monitoring), and tertiary intervention (specialized treatment), with emphasis on establishing effective linkages between schools and community/specialty hospitals to address unidirectional referral limitations. (3) “School-sport” integration: International and national strategies rarely mention in-depth cooperation between schools and sports professional organizations. We propose inviting sports experts into schools for guidance, professional training of physical education teachers for intervention delivery, and designing specialized AIS prevention exercises that incorporate traditional Chinese sports (Tai Chi, YiJinJing, etc.) with proven spinal health benefits. This represents a novel contribution to international prevention strategies. This study systematically integrates these three aspects to form a comprehensive prevention and control network that is superior to single intervention models. Figure 2 illustrates the strategy of preventing AIS through physical activity in the school perspective.

### 4.5 Innovations and limitations

The systematic review included literature published between 2015 and 2025, representing the latest progress in the field with current timeliness and reference value. This paper constructs a multidimensional analysis framework examining the relationship between physical activity and AIS across three dimensions: activity types (recreational, competitive, traditional Chinese, and school sports), intensity thresholds, and capacity changes, filling research gaps through comprehensive analysis. Additionally, this paper analyzes the unique value of traditional Chinese sports (Tai Chi and YiJinJing) for AIS prevention. This paper also proposes a novel trinity prevention system integrating “school-family”, “school-medicine”, and “school-sport” approaches, providing scientific basis for precise school-based prevention strategies.

There are still some limitations in this paper: (1) Study design limitations: Due to limited global research, most included studies were cross-sectional (*n* = 11), with fewer cohort (*n* = 2), case-control (*n* = 5), and RCT studies (*n* = 3), which limits causal inference and evidence strength. (2) Heterogeneity issues: The included literature covered diverse physical activities, content, and designs, resulting in high heterogeneity that prevented meta-analysis for accurate effect size estimation. (3) Search strategy limitations: The search process excluded some non-compliant literature, which may lead to a certain degree of selection bias and affect the comprehensiveness of the conclusions. (4) Varying methods of physical activity assessment: The diverse physical activity assessment methods in included studies (questionnaires, accelerometers, self-reports) and lack of uniform standards may affect result comparability. (5) Limitations of diagnostic criteria: A small number of studies used the Adams test to diagnose scoliosis, which, although highly sensitive and specific, is not the gold standard for diagnosing AIS and may affect the accuracy of disease definition. (6) Uneven distribution of literature: The geographical distribution of the included literature was uneven, and it was mainly from Europe, America and East Asia, so there may be geographical bias. (7) Inadequate explanation of potential mechanisms: The elaboration of how physical activity affects the development of AIS through biomechanical and physiological mechanisms is relatively limited, and in-depth analysis of pathophysiological mechanisms is lacking.

## 5 Conclusion

Physical activity is closely related to AIS, with varied findings across literature. Most recreational and traditional Chinese sports can prevent AIS development, while prolonged engagement in competitive sports, dance, and artistic gymnastics increases AIS risk. It is not clear whether unilateral exercise causes and exacerbates AIS, and more authoritative studies are needed to prove it. Adolescents need at least 1 hour of daily physical activity to effectively prevent AIS, while optimal thresholds remain undetermined. Adolescents with established AIS are less physically active than healthy adolescents and require more guidance and education. Schools should build a multi-dimensional AIS prevention system by increasing the daily physical activity time, optimizing the physical education curriculum, and combining family and medical resources. Schools should promote integrated “school-family”, “school-medicine” and “school-sport” approaches to create favorable environments for physical activities and establish comprehensive scoliosis prevention systems.

## Data Availability

The original contributions presented in the study are included in the article/Supplementary Material, further inquiries can be directed to the corresponding author.
