# Supplementary material for: Protective and risk physical activities for adolescent idiopathic scoliosis: a systematic review identifying one-hour daily activity threshold and Chinese school-based prevention framework
Source: Front Sports Act Living. 2025 Sep 2;7:1644314. doi: 10.3389/fspor.2025.1644314 (PMC12441802; doi:10.3389/fspor.2025.1644314)
Supplement: Supplementary file 1 [file Datasheet1.docx]

**SUPPLEMENTARY MATERIALS 1**

Contents:

Table S1. Quality assessment results for case-control and cohort studies (NOS scores)

Table S2. Quality assessment results for cross-sectional studies (AHRQ scores)

Figure S1. Risk of bias graph for randomized controlled trials

Figure S2. Risk of bias summary for randomized controlled trials

Table S3. Table S3. Jadad Scale Quality Assessment for Randomized Controlled Trials

**Table S1. Quality assessment results for case-control and cohort studies using Newcastle-Ottawa Scale (NOS)**

| **Study** | **Selection** | **Comparability** | **Exposure** | **Scores** | **Quality** |
| --- | --- | --- | --- | --- | --- |
| McMaster (2015) | 3 | 2 | 3 | 8 | H |
| Tobias  (2019) | 4 | 2 | 3 | 9 | H |
| Chopra (2020) | 3 | 2 | 2 | 7 | H |
| Negrini (2023) | 3 | 2 | 3 | 8 | H |
| Gaume  (2020) | 4 | 2 | 2 | 8 | H |
| Diarbakerli (2016) | 4 | 2 | 3 | 9 | H |
| de Assis (2021) | 3 | 2 | 2 | 7 | H |

High quality: H, Moderate quality: M, Low quality: L

**Table S2. Quality assessment results for cross-sectional studies using AHRQ methodology checklist**

| **Study** | **1** | **2** | **3** | **4** | **5** | **6** | **7** | **8** | **9** | **10** | **11** | **Scores** | **Quality** |
| --- | --- | --- | --- | --- | --- | --- | --- | --- | --- | --- | --- | --- | --- |
| Watanabe (2017)^[28]^ | √ | √ | √ | √ | √ | √ |  |  |  | √ | √ | 8 | H |
| Scaturro (2021)^[10]^ | √ | √ | √ | √ | √ |  |  |  |  |  | √ | 6 | M |
| Steinberg (2021)^[29]^ | √ | √ | √ | √ | √ | √ | √ |  |  |  | √ | 8 | H |
| Zain (2016)^[30]^ | √ | √ | √ | √ | √ |  |  |  |  | √ | √ | 7 | M |
| Zain (2015)^[31]^ | √ | √ | √ | √ | √ |  | √ |  |  |  |  | 6 | M |
| González-Ruiz (2025)^[32]^ | √ | √ | √ | √ | √ | √ | √ |  |  | √ | √ | 9 | H |
| Zhao (2023)^[9]^ | √ | √ | √ | √ | √ | √ |  |  |  |  | √ | 7 | M |
| Zhang (2023)^[33]^ | √ | √ | √ | √ | √ | √ |  |  |  |  | √ | 7 | M |
| Yu  (2023)^[34]^ | √ | √ | √ | √ | √ |  |  |  |  |  | √ | 6 | M |
| Huang (2024)^[35]^ | √ | √ | √ | √ | √ | √ |  |  |  |  | √ | 7 | M |
| Glavaš (2023)^[11]^ | √ | √ | √ | √ | √ |  | √ |  |  | √ | √ | 8 | H |

High quality: H, Moderate quality: M, Low quality: L, 1: Define the source of information (survey, record review). 2: List inclusion and exclusion criteria for exposed and unexposed subjects (cases and controls) or refer to previous publications. 3: Indicate time period used for identifying patients. 4: Indicate whether or not subjects were consecutive if not population-based. 5: Indicate if evaluators of subjective components of study were masked to other aspects of the participants. 6: Describe any assessments undertaken for quality assurance purposes (e.g., test/retest of primary outcome measurements). 7: Explain any patient exclusions from analysis. 8: Describe how confounding was assessed and/or controlled. 9: If applicable, explain how missing data were handled in the analysis. 10: Summarize patient response rates and completeness of data collection. 11: Clarify what follow-up, if any, was expected and the percentage of patients for which incomplete data or follow-up was obtained.


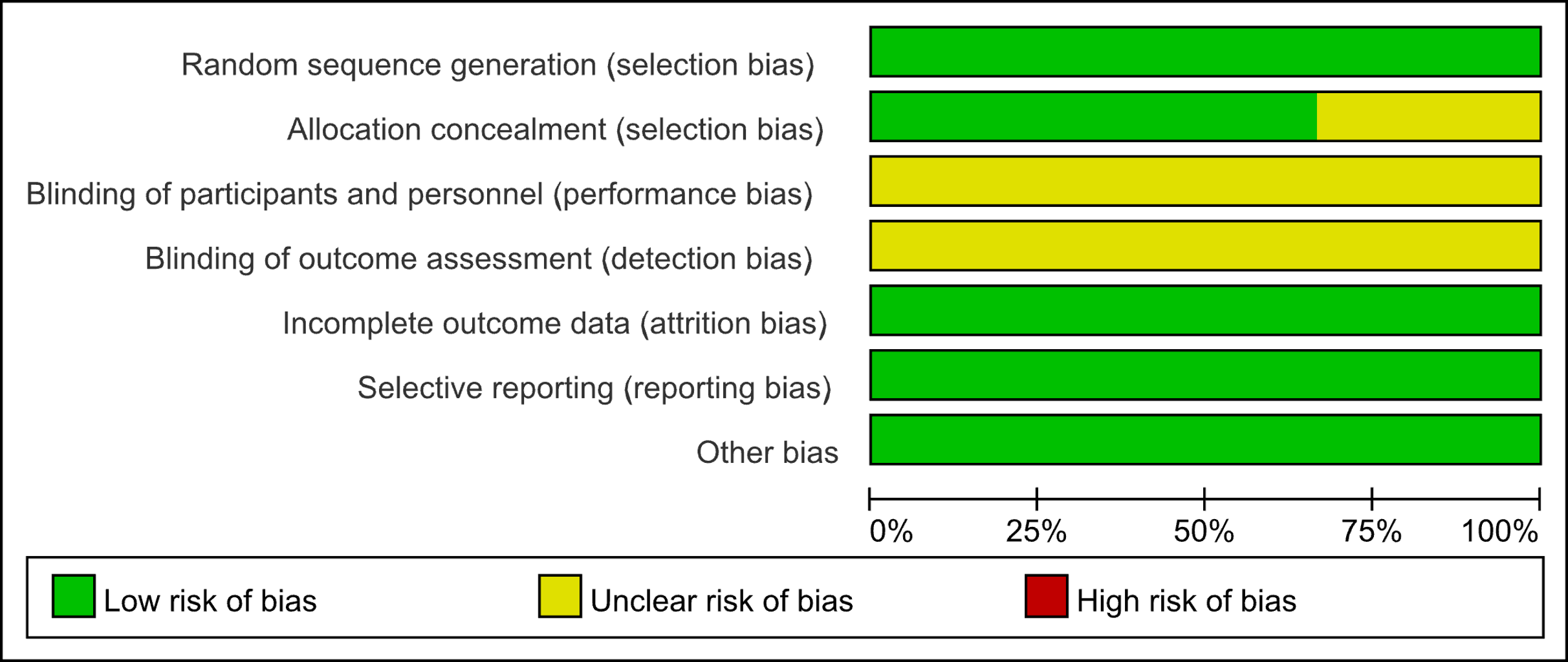


**Figure S1. Risk of bias graph for randomized controlled trials**


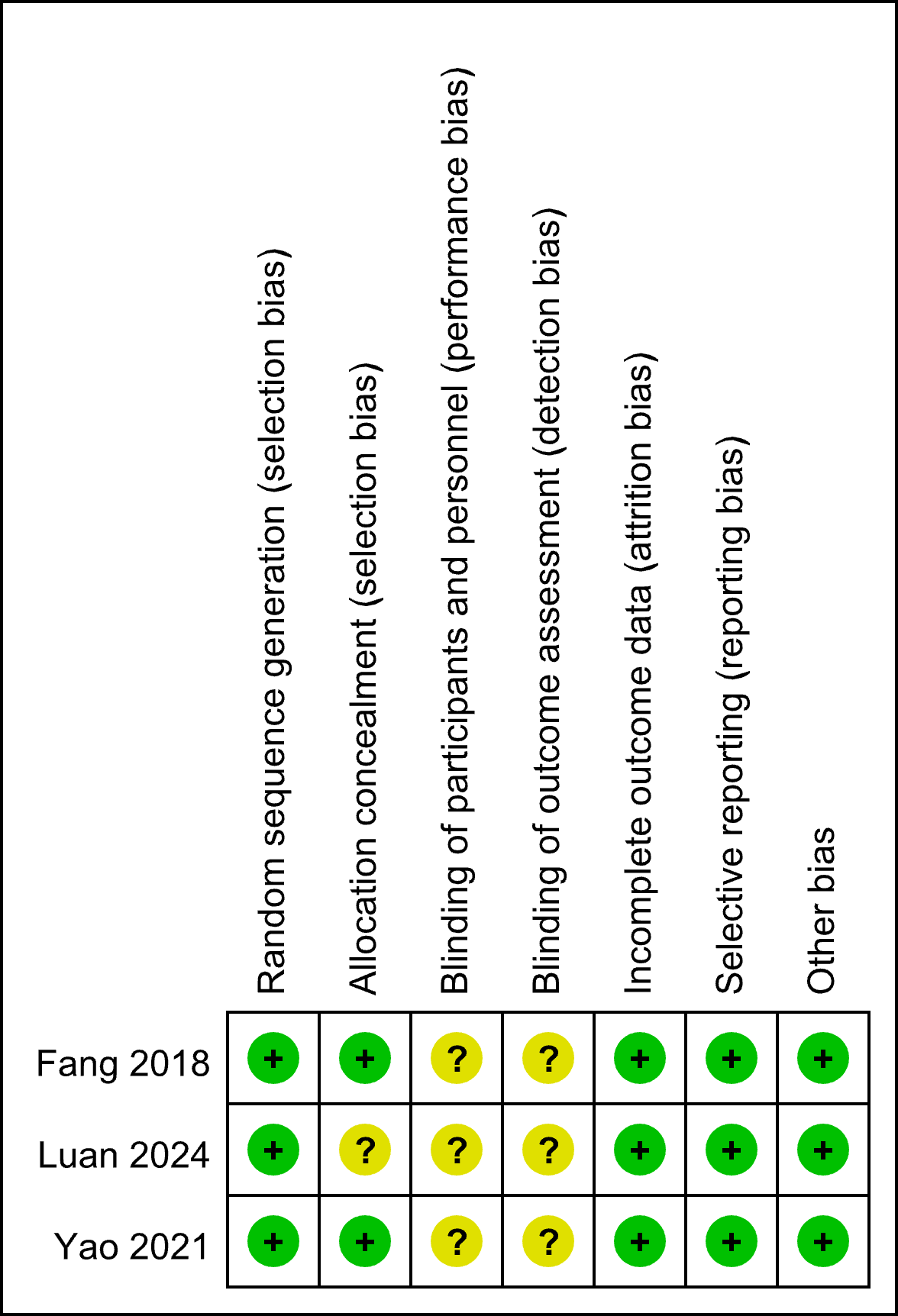


**Figure S2. Risk of bias summary for randomized controlled trials**

**Table S3. Jadad Scale Quality Assessment for Randomized Controlled Trials**

| **Study** | **1** | **2** | **3** | **4** | **5** | **6** | **7** | **Scores** | **Quality** |
| --- | --- | --- | --- | --- | --- | --- | --- | --- | --- |
| Fang (2018) | 1 | 1 | 1 | 0 | 1 | 0 | 0 | 4 | H |
| Luan  (2024) | 1 | 0 | 1 | 1 | 1 | 0 | 0 | 3 | H |
| Yao (2021) | 1 | 0 | 1 | 1 | 1 | 0 | 0 | 4 | H |

High quality: H, Low quality: L. 1: Was the study described as randomized? 2: Was the method used to generate the sequence of randomisation described and appropriate? 3: Was the study described as double blind? 4: Was the method of double blinding described and appropriate? 5: Was there a description of withdrawals and dropouts? 6: Deduct one point if the method used to generate the sequence of randomisation was described and it was inappropriate. 7: Deduct one point if the study was described as double blind but the method of blinding was inappropriate. The possible score range is 0–5, with 3–5 and 0–2 scores indicating high and low qualities, respectively.
